# Mitochondria and the Actin Cytoskeleton in Neurodegeneration

**DOI:** 10.1002/cm.70095

**Published:** 2026-01-08

**Authors:** Shivani Tuli, Preet Patel, Aneri Shethji, David Gau

**Affiliations:** ^1^ Department of Bioengineering University of Pittsburgh Pittsburgh Pennsylvania USA; ^2^ Media Arts and Sciences, MIT Cambridge Massachusetts USA; ^3^ Department of Pathology University of Pittsburgh Pittsburgh Pennsylvania USA

**Keywords:** actin cytoskeleton, mitochondria dysfunction, mitochondria‐cytoskeleton crosstalk, neurodegeneration

## Abstract

Mitochondrial dysfunction and cytoskeletal disorganization are widely recognized hallmarks of neurodegenerative diseases such as Alzheimer's disease (AD), Parkinson's disease (PD), Huntington's disease (HD), and amyotrophic lateral sclerosis (ALS). Although these disorders differ in clinical presentation and etiology, accumulating evidence points to a shared cellular vulnerability at the intersection of mitochondrial dynamics and actin cytoskeletal regulation. In this review, we examine the emerging role of actin–mitochondria crosstalk as a convergent mechanism in neurodegeneration. We discuss how disruptions in actin filament remodeling, mitochondrial fission and fusion, organelle transport, and mitophagy contribute to neuronal dysfunction and loss across these diseases. Particular attention is given to disease‐specific pathways, including cofilin‐actin rod formation in AD, α‐synuclein–driven actin disruption in PD, mutant huntingtin's effects on mitochondrial fragmentation in HD, and profilin‐1–associated mitochondrial defects in ALS. By synthesizing findings from diverse models, we highlight how perturbations in the cytoskeleton–mitochondria interface may act as an upstream trigger and amplifier of neurodegenerative cascades. We also outline key knowledge gaps and propose future directions for research, with an emphasis on targeting actin–mitochondrial interactions as a potential therapeutic strategy across multiple neurodegenerative conditions.

## 1 Introduction

Neurodegenerative disorders such as Alzheimer's disease (AD), Parkinson's disease (PD), Huntington's disease (HD), and amyotrophic lateral sclerosis (ALS) represent a growing global health burden. AD, the most common cause of dementia, currently afflicts over 6.9 million Americans, a number expected to double by 2060 as populations age (“2024 Alzheimer's Disease Facts and Figures,” 2024). PD is the second most prevalent neurodegenerative disease after AD, affecting roughly 1% of individuals over the age of 60 (Zafar and Yaddanapudi 2025). HD and ALS are less common but equally devastating: HD has a prevalence of up to 2.7 per 100,000 individuals (Ajitkumar and De Jesus 2025) and ALS, while relatively rare, is projected to increase from ~222,000 global cases in 2015 to nearly 377,000 by 2040 due to population aging (Arthur et al. 2016). Despite their diverse clinical presentations, memory loss in AD, movement disorders in PD and HD, and motor paralysis in ALS, these diseases share striking convergent mechanisms at the cellular level.

Mitochondrial dysfunction and cytoskeletal abnormalities (especially involving the actin network) are central pathological features across AD, PD, HD, and ALS. Mitochondria in affected neurons show impaired energy metabolism, excessive oxidative stress, and dysregulated dynamics (fragmentation and transport deficits), while the neuronal cytoskeleton exhibits derangements such as actin filament aggregation, impaired organelle trafficking, and synaptic structural defects. Crucially, recent evidence implicates the intimate crosstalk between mitochondrial dynamics and the actin cytoskeleton as a unifying thread in neurodegeneration (Johansson et al. 2015; Li et al. 2004; Spinazzi et al. 2012; Subramaniam and Chesselet 2013; Taylor et al. 2016; Yang 2025). Here, we review how disruptions in mitochondrial–actin interactions contribute to the pathogenesis of AD, PD, HD, and ALS, focusing on the mechanisms of mitochondrial fission/fusion imbalance, transport defects, and mitophagy failure. We highlight disease‐specific examples, from amyloid‐induced cofilin‐actin rods in AD to α‐synuclein's perturbation of actin in PD, mutant huntingtin's effects on fission in HD, and profilin‐1 abnormalities in ALS, to illustrate how each disorder converges on the nexus of mitochondrial dynamics and the actin cytoskeleton. We define “actin–mitochondria crosstalk” as local actin assembly on the outer mitochondrial membrane (OMM), and mitochondria associated membrane (MAM) at mitochondria–endoplasmic reticulum contact sites (MERCs) that governs (i) Drp1‐dependent fission site initiation and constriction, (ii) short‐range positioning and microtubule‐to‐actin hand‐off via myosins, (iii) Ca^2+^ microdomain formation and transfer at MAMs for calcium homeostasis and lipid metabolism, and (iv) actin‐enabled mitophagy and organelle quality control (Bingol and Sheng 2016; Borbolis and Palikaras 2022; Hatch et al. 2016). In neurons, this tripartite interaction is particularly important for calcium buffering and ATP production at sites of high energy demand (Proulx et al. 2021). Prior work has broadly covered mitochondrial dysfunction or the neuronal cytoskeleton. Our focus is disease‐centric and mechanism‐first: we map specific disease drivers like Aβ/tau (AD), α‐syn/LRRK2 (PD), mHTT (HD), and PFN1/C9ORF72 (ALS) onto the four checkpoints above. We show how disruption at each node yields shared outcomes (hyperfission, mislocalization, impaired mitophagy) through causal actin‐dependent steps rather than association alone. We further integrate instances where actin pharmacology or genetics rescues mitochondrial phenotypes, highlighting the therapeutic tractability of the actin‐mitochondria axis.

**FIGURE 1:**
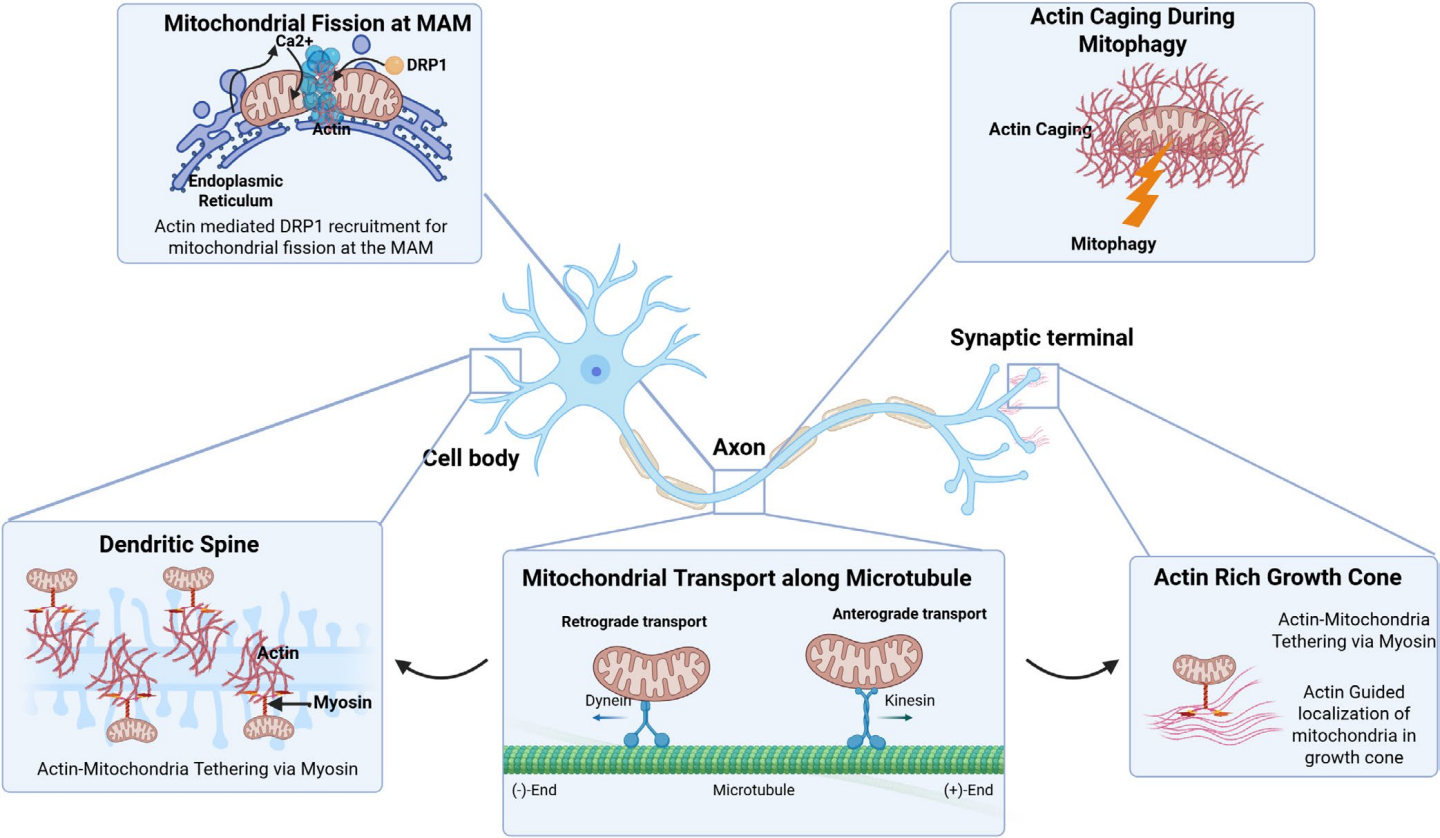
Actin–mitochondria interactions regulate neuronal function and homeostasis. Neurons rely on coordinated interactions between the actin cytoskeleton and mitochondria to maintain bioenergetic homeostasis, synaptic function, and intracellular organization. Shown here are key subcellular contexts where actin–mitochondria crosstalk is critical. Actin filaments facilitate the recruitment of Drp1 to the MAM and mediate fission in a Ca^2+^ dependent manner. During mitophagy, damaged mitochondria are encaged by actin filaments to promote their isolation and degradation. In dendritic spines, actin filaments tether mitochondria via myosin motors, ensuring localized energy support for synaptic activity. Mitochondria are actively transported along microtubules, with dynein mediating retrograde and kinesin mediating anterograde transport; actin is involved in anchoring and positioning mitochondria at specific destinations. In axonal growth cones, actin filaments and myosin coordinate the precise localization of mitochondria, supporting growth and guidance cues.

Neurons are characterized by their highly polarized morphology and substantial energetic requirements, necessitating a robust mitochondrial network capable of efficient ATP synthesis, calcium homeostasis, and regulated apoptotic signaling, coupled with a dynamic cytoskeleton that supports structural integrity, organelle trafficking, and synaptic plasticity (Borbolis and Palikaras 2022; Giampetruzzi et al. 2019). To fulfill these critical functions, neuronal mitochondria continuously engage in dynamic remodeling processes, including cycles of fission and fusion, thereby maintaining mitochondrial quality control, optimizing bioenergetic performance, and adapting to spatially variable energy demands (Lee et al. 2016; Yadav et al. 2022). Mitochondrial positioning within neuronal compartments is meticulously regulated through active transport along microtubule‐based cytoskeletal tracks, ensuring precise mitochondrial delivery to synapses and axonal termini with high energetic demands (Figure 1 summarizes the overall connection between cytoskeleton and mitochondria). Furthermore, selective degradation of dysfunctional mitochondria through mitophagy serves as an essential homeostatic mechanism to preserve neuronal viability and prevent the deleterious effects of impaired mitochondrial function (Bingol and Sheng 2016; Yadav et al. 2022). Crucially, these dynamic mitochondrial behaviors are orchestrated in concert with the cytoskeleton. Microtubules serve as the long‐range “highways” for mitochondrial transport, whereas filamentous actin (F‐actin) forms local networks and scaffolds that guide mitochondrial positioning and division (Huang et al. 2023; Wang et al. 2018). Actin filaments and their associated actin‐binding proteins can tether mitochondria and facilitate processes such as Drp1‐mediated fission, as well as the formation of actin‐rich structures that aid in isolating damaged mitochondria during mitophagy (Moore et al. 2016; Morlino et al. 2014; Nishimura et al. 2021; Yadav et al. 2022). Therefore, a deeper understanding of how mitochondrial dynamics interface with the actin cytoskeleton is critical for elucidating the progression of age‐related neurodegeneration. The cytoskeleton is critical for maintaining dendritic spine structure, growth cone motility, and local trafficking of organelles and vesicles. Concomitantly, proper localization and movement of mitochondria within neuronal processes depend on cytoskeletal tracks and motor proteins (Mandal and Drerup 2019; Munoz‐Lasso et al. 2020; Rangaraju et al. 2019; Saxton and Hollenbeck 2012; Wegorzewska et al. 2009). It is increasingly evident that disruptions in actin dynamics can precipitate mitochondrial mislocalization or dysfunction, and vice versa, linking these two cellular systems in the early steps of neurodegeneration (Theunissen et al. 2021). Table 1 summarizes the key findings discussed below for the various neurodegenerative diseases.

**TABLE 1:** Summary of mitochondria and cytoskeletal effects in neurodegenerative diseases.

Process	AD	PD	HD	ALS
Mito fission/fusion	↑ fission (Drp1, tau)	↑ fission (Drp1, α‐syn)	↑ fission (Drp1, mHTT)	↓ fusion, ↑ fission^a^
Transport defect	Aβ‐induced cofilin‐actin rods	α‐syn‐spectrin‐actin disruption	mHTT‐actin‐motor block	Cofilin‐actin rods, KIF5A loss
Actin aggregate	Cofilin‐actin rods	α‐syn, LRRK2‐F‐actin	mHTT binds F‐actin	PFN1 mutations, cofilin‐actin rods
Mitophagy disruption	Impaired recruitment (cofilin, actin dynamics)	LRRK2, PINK1/Parkin pathway	Unknown	PFN1, C9ORF72 dysfunction

*Note*: Dark grey highlights indicate shared Drp1‐mediated fission in AD/PD/HD. Light grey highlights indicate shared cofilin‐actin rods in AD/ALS.

^a^ Mitochondrial fragmentation is observed in ALS motor neurons, however the cause of this result, whether from increased fission, reduced fusion, or other mechanisms is not explicitly certain.

## 2 Mitochondria–Actin Crosstalk in Alzheimer's Disease

AD is the most common form of dementia, characterized by progressive memory loss and cognitive decline. Its hallmark pathologies are extracellular amyloid‐β (Aβ) plaques and intracellular neurofibrillary tangles composed of hyperphosphorylated tau protein (De Strooper and Annaert 2000; Kang et al. 2011; Koo and Squazzo 1994). These protein aggregates disrupt neuronal function and trigger widespread synapse loss and neuronal death. Mitochondrial dysfunction has long been observed in AD patients, including deficits in cerebral glucose metabolism and respiratory chain activity. In parallel, cytoskeletal abnormalities, most famously neurofibrillary tangles of tau, are a central feature of AD. While tau primarily associates with microtubules, growing evidence implicates the actin microfilament network in AD pathogenesis as well (Chua et al. 2003; Kang et al. 2011).

Synaptic dysfunction in AD is tightly linked to actin dysregulation. Aβ oligomers, which are synaptotoxic, can induce aberrant remodeling of the spine actin cytoskeleton. Notably, compounds that either stabilize F‐actin (e.g., jasplakinolide) or depolymerize actin (e.g., cytochalasin D, latrunculin A) both attenuate Aβ‐induced neurotoxicity in cellular models. This paradoxical result indicates that it is the dynamic turnover of actin filaments, excessive remodeling triggered by Aβ binding to neurons that is required for Aβ's toxic effect. Aβ can bind to cell‐surface integrin receptors, aberrantly activating focal adhesion signaling and cofilin, an actin‐depolymerizing factor (Kang et al. 2011). Downstream of Aβ, cofilin is known to accumulate in rod‐like actin aggregates in AD neurons. These cofilin–actin rods form under stress conditions and sequester actin monomers, essentially freezing local actin dynamics (Ishikawa‐Ankerhold et al. 2021). In AD brain and models, cofilin–actin rods are prevalent in dystrophic neurites and dendritic spines, where they disrupt synaptic function. Intriguingly, cofilin–actin rods can also bind and sequester hyperphosphorylated tau. The presence of tau within actin rods suggests a convergence of cytoskeletal pathologies: Aβ‐induced actin disruptions may facilitate tau mislocalization and aggregation in dendrites (Woo et al. 2019). Thus, actin abnormalities not only mediate Aβ toxicity but might also promote tau pathology, compounding synaptic damage in AD.

Mitochondria in AD neurons are fragmented, energetically compromised, and often mislocalized. Excessive mitochondrial fission has been reported in AD, partly attributed to Aβ and phosphorylated tau elevating cytosolic calcium and oxidative stress. One emerging mechanistic link between actin and mitochondria in AD is through cofilin. Under oxidative stress, a condition prevalent in AD brains, cofilin becomes aberrantly activated and translocates from the cytosol into mitochondria. Cofilin contains several cysteine residues that can be oxidized, promoting its mitochondrial import. Once inside, cofilin associates with the inner mitochondrial membrane, inducing swelling of mitochondria, loss of membrane potential, and release of cytochrome c by opening the permeability transition pore. This cofilin‐mediated mitochondrial apoptosis pathway operates independently of Bax and other canonical apoptotic factors (Chua et al. 2003; Kang et al. 2011; Klamt et al. 2009). In AD, Aβ exposure is known to induce cofilin activation and rod formation; it is plausible that some fraction of cofilin also redistributes to mitochondria in affected neurons, directly triggering cell death pathways. Beyond cofilin, disrupted actin dynamics may affect mitochondrial fission–fusion balance. Actin filaments help recruit Drp1 (dynamin‐related protein 1) to mitochondrial fission sites (Hatch et al. 2016). If Aβ or tau pathology leads to actin disorganization, Drp1 recruitment or oligomerization at mitochondria could be perturbed, contributing to the fragmented mitochondria observed in AD neurons. Indeed, mislocalization of Drp1 has been noted because of actin dysregulation in several neurodegenerative models (DuBoff et al. 2012). The result is a vicious cycle: Aβ and tau cause actin derangements that impair mitochondrial function, and failing mitochondria produce reactive oxygen species and ATP shortages that further destabilize actin polymerization, ultimately driving synaptic failure and neuron loss.

## 3 Mitochondria–Actin Interactions in Parkinson's Disease

PD is the second most common neurodegenerative disorder, marked by bradykinesia, rigidity, and tremor due to degeneration of dopaminergic neurons in the substantia nigra. Its neuropathological signature is the Lewy body, an intraneuronal inclusion enriched in α‐synuclein protein. Mitochondrial dysfunction is a central theme in PD pathogenesis: inhibitors of mitochondrial Complex I can induce PD‐like syndromes, and several familial PD genes (PINK1, Parkin, DJ‐1) directly impinge on mitochondrial quality control (Truban et al. 2017). Alongside mitochondrial deficits, cytoskeletal abnormalities have been observed in PD. While much focus has been on microtubules (e.g., disrupted axonal transport) and intermediate filaments (Lewy bodies contain neurofilament components), recent studies highlight the critical involvement of the actin microfilament system in PD pathology.

Converging genetic and experimental evidence links α‐synuclein to actin dysfunction in PD. In a Drosophila model of α‐synucleinopathy, overexpressed human α‐synuclein reorganization of the actin cytoskeleton was identified as a key mediator of neurodegeneration. Specifically, α‐synuclein was found to bind to spectrin, an actin‐binding cytoskeletal protein, and disrupt spectrin's normal function. Spectrin helps stabilize the cortical actin network; when α‐synuclein binds and perturbs spectrin, it leads to F‐actin reorganization and excessive actin polymerization in neurons (Ordonez et al. 2018). Ordoñez et al. demonstrated that this α‐synuclein‐induced actin reorganization has dire consequences: Drp1 mislocalization, impaired mitochondrial dynamics, and neuronal death. In postmortem PD brains and α‐syn transgenic mice, similar accumulations of F‐actin and signs of spectrin disruption have been observed, suggesting that the fly model recapitulates a fundamental disease process. Thus, α‐synuclein toxicity in PD may arise not only from protein aggregation per se, but also from a cascade of cytoskeletal disturbances leading to mitochondrial dysfunction. Supporting this notion, genetic restoration of spectrin in the α‐synuclein fly model rescued actin organization and neuronal viability (Maor et al. 2023), highlighting spectrin–actin as a therapeutic target in synucleinopathies.

Another PD‐linked protein, leucine‐rich repeat kinase 2 (LRRK2), further underscores the actin–mitochondria axis in PD. Mutations in *LRRK2* are a common cause of familial PD, and intriguingly, LRRK2 has been shown to bind and sever actin filaments. In cellular models, wild‐type LRRK2 can directly cut F‐actin, whereas PD‐associated mutant LRRK2 (e.g., G2019S) loses this actin‐severing activity and instead promotes excess stabilization of F‐actin (Bardai et al. 2018). The consequence is reminiscent of the α‐synuclein scenario: excess stabilized F‐actin leads to mislocalization of Drp1 and defective mitochondrial fission (Maor et al. 2023). Bardai et al. found that altering LRRK2 levels in neuron models influences filamentous actin content and that reducing F‐actin levels (using actin depolymerizing drugs) could rescue neurodegeneration in LRRK2 and tau transgenic fly models. Strikingly, their work connected LRRK2 and tau pathologies through an actin‐dependent mechanism and furthermore linked this pathway to PINK1 and Parkin, two other PD proteins that govern mitochondrial integrity (Bardai et al. 2018). PINK1/Parkin normally promote removal of damaged mitochondria (mitophagy); disruptions in actin dynamics might hinder this process by affecting the recruitment of autophagy machinery to mitochondria. Indeed, actin polymerization is required for efficient mitophagosome formation around mitochondria in some contexts, and LRRK2 dysfunction or α‐synuclein aggregates could impede such cytoskeletal remodeling.

Collectively, the evidence indicates that actin cytoskeletal dysregulation is not a bystander but a contributor to PD pathogenesis. α‐Synuclein and LRRK2, two disparate PD proteins, both converge on actin dynamics and consequently derail mitochondrial homeostasis. Dopaminergic neurons, with their extensive axonal arbors, are especially vulnerable to disturbances in organelle transport and energy supply. In PD models, mitochondrial movement in axons is slowed, and this could be exacerbated by actin disturbances in growth cones or presynaptic terminals where actin normally anchors mitochondria. Moreover, toxic gain‐of‐function in actin regulators (spectrin, cofilin, etc.) might activate mitochondrial death pathways analogous to those in AD. Further research is needed to clarify how early in PD pathogenesis these cytoskeletal disruptions occur and whether they represent a point of no return for neuronal survival or a reversible, druggable phenomenon.

## 4 Mitochondria–Actin Interactions in Huntington's Disease

HD is an inherited autosomal dominant neurodegenerative disease caused by an expanded CAG repeat in the *HTT* gene, encoding an abnormal polyglutamine tract in the huntingtin protein. Clinical features include motor dysfunction, including chorea, cognitive decline, and psychiatric symptoms, underpinned by selective death of medium spiny neurons in the striatum. Mutant huntingtin (mHTT) is prone to misfolding and aggregation, forming intraneuronal inclusions. Mitochondrial abnormalities have been extensively documented in HD, including impaired respiratory capacity, increased oxidative stress, and disrupted mitochondrial trafficking in neurons (Reddy et al. 2009; Shirendeb et al. 2012). Cytoskeletal involvement in HD pathology is also evident: mHTT aggregates can bind to and disturb elements of both the microtubule and actin networks. Normal huntingtin participates in axonal transport by interacting with motor proteins and vesicle trafficking complexes; its mutation leads to breakdown of these processes. mHTT derails actin–mitochondria crosstalk through three interlocking mechanisms: (i) it biases actin‐primed, Drp1‐dependent fission toward pathological fragmentation; (ii) it disrupts long‐range transport and actin‐dependent local anchoring/hand‐off of mitochondria; and (iii) it perturbs profilin/cofilin pathways, creating a feed‐forward loop that links actin stress to mitochondrial injury. Together this yields fragmented, mislocalized mitochondria with reduced ATP output and Ca^2+^ buffering at synapses, amplifying neuronal vulnerability.

In healthy neurons, short‐lived actin assemblies on mitochondria and at ER‐mitochondria contact sites help recruit and scaffold Drp1 to execute fission (Hatch et al. 2016; Moore et al. 2016). In HD models and tissue, mHTT associates with Drp1, increases Drp1 GTPase activity, and drives excessive fission, producing a fragmented mitochondrial network and bioenergetic compromise; pharmacologic or genetic dampening of Drp1 partially rescues these phenotypes (Reddy 2014; Shirendeb et al. 2012). Given actin's role in seeding fission sites, mHTT‐induced alterations to local F‐actin are poised to mis‐time or mis‐place Drp1 assembly, compounding the hyperfission tendency (Hatch et al. 2016). In cell and animal models, aggregated polyglutamine stretches have been observed to physically associate with F‐actin, potentially sequestering actin or altering its polymerization. Consequently, mitochondria in HD models become abnormally clustered with reduced processive movement, undermining distal ATP supply and Ca^2+^ buffering (Reddy et al. 2009). This transport defect is partly due to microtubule‐based transport disruption (since huntingtin interacts with microtubule motors), but actin may also play a role in the short‐range positioning of mitochondria at synapses. Interactions of mHTT with actin and actin‐binding proteins can lead to actin network disturbances that impede the movement of mitochondria and other organelles. Further, at synapses and growth cones, actin–myosin systems mediate microtubule‐to‐actin hand‐off and local anchoring of mitochondria; when actin organization is perturbed, these short‐range mechanisms fail and compound microtubule‐based transport deficits (Mandal and Drerup 2019). Indeed, in vitro experiments demonstrate that misfolded huntingtin fragments can cross‐link actin filaments and alter actin polymerization dynamics (Singh et al. 2024). These cytoskeletal perturbations, combined with direct effects of mHTT on motor proteins (like dynein/dynactin and kinesin) (Bossy‐Wetzel et al. 2008; Caviston et al. 2007), result in a failure of mitochondria to properly supply the distal parts of neurons with energy, contributing to synaptic dysfunction and neuronal vulnerability.

Furthermore, the cellular energy deficit caused by mHTT (via transcriptional dysregulation of metabolic genes and direct mitochondrial damage [Li et al. 2010]) can trigger compensatory actin responses. Energy stress often stabilizes F‐actin (as cells attempt to conserve ATP by reducing actin turnover), and this could feed back to worsen transport and fission abnormalities. HD models have demonstrated that restoring normal mitochondrial fission/fusion dynamics or enhancing mitochondrial transport can alleviate neuronal degeneration (Shirendeb et al. 2012), underscoring how critical these processes are. However, if upstream actin pathology is not addressed, such interventions may be only partially effective.

HD pathology involves a multifaceted disruption of intracellular order: mutant huntingtin deranges the cytoskeleton and mitochondria in tandem. Actin filaments, together with microtubules, form a scaffold for organelle movement; mHTT undermines both, leading to “traffic jams” of mitochondria and vesicles in axons (Vitet et al. 2020). The ensuing energy deprivation and calcium mishandling further injure neurons. A particularly insidious aspect of HD is that the cause is monogenic, mHTT, yet it produces a cascade of secondary defects. This makes it challenging to untangle cause from effect in mitochondria‐actin phenotypes. Are actin aggregates in HD a direct toxic effect of mHTT sequestering actin, or an indirect result of energy failure and oxidative stress? Evidence suggests both contribute: mHTT can directly bind cytoskeletal proteins, and HD neurons also experience chronic metabolic stress that promotes cytoskeletal oxidation and protein cross‐linking. While energy failure and oxidative stress further perturb actin (e.g., cofilin oxidation), the causal chain above: mHTT causing actin‐primed Drp1 hyperfission, transport/anchoring failure and cofilin/profilin dysregulation provides a mechanistic framework for the observed mitochondrial fragmentation and synaptic vulnerability. What is clear is that interventions targeting only one side of the problem (solely mitochondria or solely cytoskeleton) may not fully rescue neuronal health; a combined approach might be necessary.

## 5 Mitochondria–Actin Interactions in Amyotrophic Lateral Sclerosis

ALS involves the progressive degeneration of upper and lower motor neurons, leading to muscle weakness, paralysis, and death typically within 2–5 years of diagnosis. Pathologically, ALS features inclusions of proteins such as TDP‐43 in neurons and astrogliosis. Unlike AD, PD, and HD, which are largely sporadic with age, a significant subset of ALS cases is familial, caused by mutations in diverse genes. Remarkably, many ALS‐linked genes encode proteins related to cytoskeletal dynamics or axonal transport (e.g., *PFN1*, *DCTN1*, *KIF5A*, *NEFH/NEFL* for neurofilament proteins, *SPAST* for spastin) (Castellanos‐Montiel et al. 2020). This underscores the importance of the cytoskeleton in motor neuron maintenance. Mitochondrial dysfunction is also a well‐documented aspect of ALS, with evidence of respiratory chain deficiencies, increased oxidative damage, and abnormal mitochondrial morphology in patient tissues and models (Smith et al. 2019). Motor neurons, given their extraordinary length, are highly dependent on efficient organelle transport and energy distribution, making them particularly susceptible to combined cytoskeletal and mitochondrial insults (Theunissen et al. 2021). In ALS, actin‐mitochondria crosstalk fails via three nodes (i) a direct mitochondrial role for PFN1 that is required for organelle homeostasis, (ii) cofilin–actin rods that both physically block neurites and, when oxidized, can translocate to mitochondria to trigger permeability transition/apoptosis, and (iii) combined long‐range transport deficits (KIF5A/dynactin) with actin–myosin hand‐off/anchoring failure at distal compartments. Together, these disruptions produce depolarized, morphologically abnormal mitochondria with reduced ATP/Ca^2+^ buffering (Castellanos‐Montiel et al. 2020; Chua et al. 2003; Kang et al. 2011; Maloney and Bamburg 2007; Mandal and Drerup 2019; McGoldrick and Robertson 2023; Read et al. 2024; Saxton and Hollenbeck 2012; Theunissen et al. 2022; Uruk et al. 2024).

Impairment of axonal transport is an early feature in many ALS models, and mitochondria are among the cargoes that become stalled. Mutations in the kinesin motor KIF5A (familial ALS) or in the dynactin complex (e.g., *DCTN1* mutation causes Perry syndrome, an ALS/PD overlap) cause deficits in both anterograde and retrograde transport of mitochondria in neurons. Even in sporadic ALS, evidence of sluggish mitochondrial movement in axons has been reported (Castellanos‐Montiel et al. 2020). Actin filaments are integral to the distal delivery of mitochondria; in growth cones and at neuromuscular junctions, myosin motors shuttle mitochondria from microtubule tracks to actin‐rich areas. Thus, disruptions in actin organization could further exacerbate mitochondrial mislocalization. Indeed, motor neuron cultures under chronic stress form cofilin–actin rods similar to those in AD, which can block transport in neurites (Maloney and Bamburg 2007; Maloney et al. 2005; Uruk et al. 2024; Wurz et al. 2022). Cofilin pathology in ALS is an area of active investigation, with some studies suggesting that ALS‐linked proteins (like the C9ORF72 repeat expansion) may induce cofilin activation and rod formation in neurons (Giampetruzzi et al. 2019). Such rods, if persistent, would impair delivery of mitochondria to synaptic terminals and hinder synaptic maintenance.

A direct link between actin regulators and mitochondrial function in ALS comes from profilin 1 (PFN1). *PFN1* mutations cause a form of familial ALS, and profilin is chiefly known as an actin monomer‐binding protein that aids in actin polymerization. However, recent work uncovered an unexpected mitochondrial role for profilin: PFN1 localizes inside mitochondria and is necessary for normal mitochondrial homeostasis. Loss of PFN1 in cells led to accumulation of depolarized, structurally abnormal mitochondria and a paradoxical increase in mitophagy, suggesting a compensatory response (Read et al. 2024), implying that profilin's function inside mitochondria (perhaps in maintaining mitochondrial actin or metabolite balance) is crucial. In ALS, PFN1 mutations might cause both cytoskeletal and mitochondrial pathology: misfolded or aggregation‐prone PFN1 could disrupt actin dynamics and directly damage mitochondria by losing its normal mitochondrial function. Other ALS‐associated proteins hint at actin–mitochondria intersections. For example, SOD1 mutants (the first discovered ALS gene) tend to increase mitochondrial ROS, which can secondarily oxidize actin and cause cytoskeletal disarray (Suthar and Lee 2023). Another example is *C9ORF72*, the most common genetic cause of ALS/FTD, which has been implicated in endosomal trafficking and autophagy; loss of C9ORF72 function may affect actin dynamics through signaling pathways, indirectly influencing mitochondrial clearance (Giampetruzzi et al. 2019). Additionally, because actin remodeling is required for mitophagosome formation and isolation of damaged mitochondria, ALS‐linked actin defects (PFN1 mutation; C9ORF72‐driven actin dysregulation) are predicted to impair mitophagy flux even when upstream PINK1/Parkin signals remain intact (Borbolis and Palikaras 2022).

The net effect in ALS is a convergence of cytoskeletal disorganization and mitochondrial dysfunction as mutually reinforcing pathologies (Theunissen et al. 2021). Electron microscopy of ALS motor neurons shows shrunken, misshapen mitochondria often accumulating in the cell body near areas of neurofilament aggregation (Castellanos‐Montiel et al. 2020). This suggests that axonal transport failure (due to cytoskeletal gene mutations or cofilin rods) causes distal parts of the axon to be deprived of mitochondria, while proximal parts suffer from cluttered organelles and protein aggregates. Energetically, motor neurons under stress cannot sustain their large axonal fields. The actin cytoskeleton, particularly at the neuromuscular junction, is critical for maintaining synaptic structures; degeneration of the neuromuscular junction in ALS may involve loss of actin scaffolding as well as withdrawal of mitochondria that normally support synaptic function. Therapeutically, attempts to stabilize cytoskeletal elements in ALS models (e.g., with tubulin‐binding drugs or actin‐modulating compounds) have shown mixed results. The challenge is that the cytoskeletal defects in ALS are diverse, affecting actin filaments, microtubules, and intermediate filaments, suggesting that broad‐spectrum or combination therapies may be required.

## 6 Shared Molecular Players Across Diseases

**FIGURE 2:**
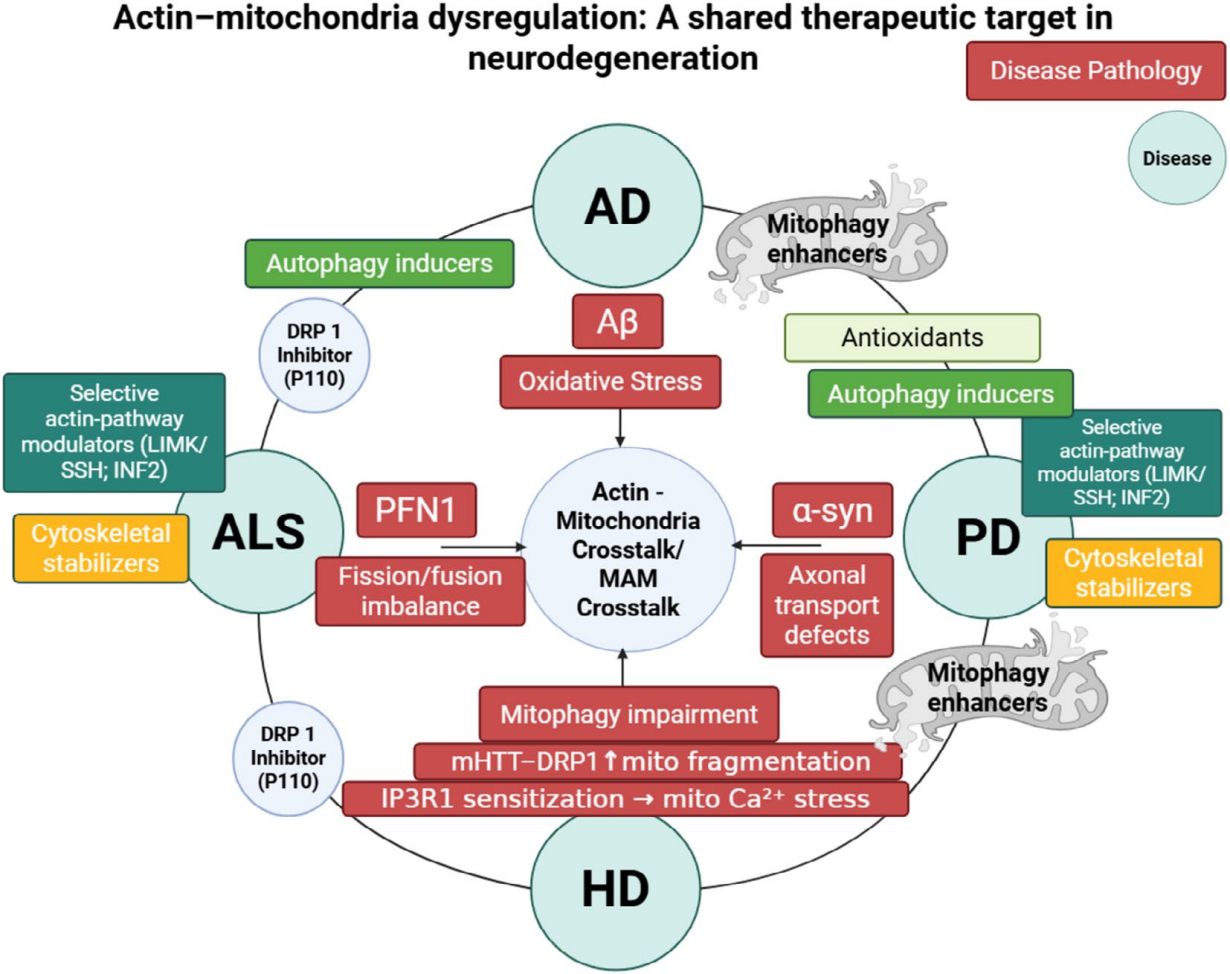
Actin–mitochondria crosstalk as a convergent pathological and therapeutic axis in neurodegenerative diseases. Disruptions in the interplay between the actin cytoskeleton and mitochondrial function represent a shared mechanism across major neurodegenerative disorders, including Alzheimer's disease, Parkinson's disease, Huntington's disease, and amyotrophic lateral sclerosis. Central pathological drivers, such as amyloid‐β and oxidative stress in AD, α‐synuclein in PD, mutant huntingtin in HD, and profilin‐1 in ALS, converge on mitochondrial–cytoskeletal dysregulation, resulting in impaired fission/fusion dynamics, axonal transport defects, and mitophagy failure. This dysfunction contributes to neuronal vulnerability and degeneration. Targeted therapeutic strategies under investigation include actin modulators, cytoskeletal stabilizers, autophagy inducers, mitophagy enhancers, antioxidants, and Drp1 inhibitors. Collectively, these approaches aim to restore cytoskeletal integrity and mitochondrial homeostasis, highlighting the actin‐mitochondria axis as a unifying and actionable target in neurodegenerative disease treatment.

Across AD, PD, HD, and ALS, a compact set of actin mitochondria regulators recurs. Drp1, the central mitochondrial fission regulator, is disrupted across all four diseases through actin‐dependent mechanisms. Disease contexts repeatedly drive Drp1 mislocalization or hyperactivation: tau/oxidative stress in AD, α‐syn–spectrin disruption in PD, and mutant huntingtin binding to Drp1 in HD, all tilt networks toward pathological fragmentation and transport failure (DuBoff et al. 2012; Hatch et al. 2016; Ordonez et al. 2018; Shirendeb et al. 2012). Cofilin emerges as a second shared node. This actin‐severing protein acts as both a driver and sensor of stress: it assembles into cofilin‐actin rods that freeze local dynamics and block axonal transport (prominent in AD and ALS). Under oxidative conditions, cofilin translocates to mitochondria to trigger depolarization and cytochrome c release (Bamburg et al. 2010; Munsie and Truant 2012; Uruk et al. 2024; Wurz et al. 2022). Disease‐linked proteins with primary identities outside the actin–mitochondria axis also converge here. α‐Synuclein perturbs spectrin/actin and thereby Drp1‐dependent fission in PD models, while PFN1, a canonical actin regulator, localizes within mitochondria where it is required for their homeostasis in ALS models (Froula et al. 2018; Ordonez et al. 2018; Read et al. 2024). Together with actin assembly at the OMM and mitochondria ER contacts that support mitophagy and calcium exchange, these factors define a conserved actin–mitochondria checkpoint (Bingol and Sheng 2016; Yadav et al. 2022). From this perspective, three recurrent failure modes dominate: (i) Drp1‐skewed fission/fusion imbalance, (ii) short‐range anchoring and hand‐off defects that mar organelle positioning at synapses and growth cones, and (iii) inefficient mitophagy initiation at OMM/MAMs. Recognizing these common nodes, Figure **2** illustrates cross‐disease biomarkers and combinatorial interventions that rebalance fission, restore local actin remodeling, and sustain mitochondrial quality control.

## 7 Targeted Therapeutic Strategies Under Investigation

### 7.1 Correcting Mitochondrial Fission–Fusion at Actin/MAM Checkpoints

The cell‐penetrant peptide P110 disrupts the stress‐induced Drp1‐Fis1 interaction (a fission path closely tied to actin/MERCs remodeling), rescuing mitochondrial membrane potential, reducing ROS, and improving neuronal viability in models relevant to AD and brain injury. Newer work in human cells shows reduced APP/BACE1 protein and improved mitochondrial readouts after P110 exposure, supporting disease‐relevant translation, although in vivo CNS efficacy still requires more proof (Srivastava et al. 2023). In contrast, the widely used small molecule mdivi‐1 is now recognized as a reversible complex‐I inhibitor and not a specific Drp1 inhibitor, so it should not be used to infer Drp1 pharmacology. Small‐molecule M1 increases mitofusin activity and promotes mitochondrial fusion; in neuronal contexts, fusion promotion is a plausible counter to pathological fission that is often nucleated at actin‐rich ER–mitochondria contacts. While most reports are preclinical, they establish a tractable chemistry space for pro‐fusion therapeutics (Au et al. 2022).

### 7.2 Restoring Actin Homeostasis Near Mitochondria

The approved vasodilator fasudil (a ROCK inhibitor) reduces Aβ/tau burden, oxidative stress, and neuroinflammation while improving cognition in AD mouse models and reducing metabolic derangements in tauopathy models (Collu et al. 2024). Because ROCK lies upstream of LIMK, ROCK inhibitors provide a credible lever on the actin–mitochondria axis, though dose and regional effects matter and clinical data in neurodegeneration remain limited. LIMK inhibitors represent another approach. LIMK1/2 inhibitors (e.g., SR‐7826) prevent Aβ42‐induced dendritic spine loss in hippocampal neurons (Henderson et al. 2019), consistent with excessive LIMK activity being deleterious; medicinal‐chemistry campaigns have recently produced highly selective tool molecules (e.g., MDI‐114215 [Baldwin et al. 2025]), creating a cleaner way to probe this node. That said, LIMK governs plasticity, and hippocampal studies indicate that the timing of inhibition can impair memory encoding, so CNS use will demand precise spatiotemporal control. Lastly, regarding cofilin‐directed strategies, because cofilin–actin rods arrest local actin dynamics and can gate mitochondrial injury, several groups have explored cofilin‐modulating peptides and small molecules (Shaw and Bamburg 2017). BBB‐permeant peptides that tune cofilin phosphorylation/activation state have shown target engagement in rodent CNS; recent small‐molecule “cofilin inhibitors” improved neurological outcomes in post‐stroke models (Almarghalani et al. 2024). For AD/ALS contexts where rods are prevalent, approaches that prevent rod nucleation (e.g., limiting oxidative stress that activates cofilin) may be more tractable than chronic cofilin inhibition.

### 7.3 Enhancing Mitophagy and Mitochondrial QC

Mitophagy boosters like urolithin A (UA) enhance PINK1/Parkin‐dependent mitophagy and improve cognition and neuropathology in multiple AD mouse models, as shown in recent 2024 reports; human safety/PK are established in other indications, but CNS disease efficacy remains to be proven (Hou et al. 2024). Parallel efforts target the deubiquitinase USP30, a negative regulator of Parkin‐mediated mitophagy; new small‐molecule USP30 inhibitors show robust target engagement and mitophagy enhancement in neurons (Okarmus et al. 2024). Anti‐ROS nanodots that restore mitochondrial potential can facilitate PINK1/Parkin mitophagy and protect dopaminergic neurons in MPTP models, thereby bridging oxidative stress control to quality‐control activation (Xia et al. 2023).

### 7.4 Stabilizing Organelle Transport to Synapses (Mitochondrial Motility Hand‐Off)

Mitochondrial transport defects are prominent across all four diseases, making restoration of organelle motility a key therapeutic target. HDAC6 inhibition (increasing α‐tubulin acetylation) restores mitochondrial motility and axonal transport in multiple neuronal disease models (Pham et al. 2025; A. S. T. Smith et al. 2022). Translationally, lactoferrin‐decorated PLGA nanoparticles carrying the HDAC inhibitor CAY10603 improved BBB delivery and reversed mitochondrial dysfunction, α‐syn accumulation, and neuroinflammation in a PD model. This exemplifies how nanocarriers can enable CNS‐targeted cytoskeletal modulation linked to mitochondrial health. Ligand‐decorated polymeric nanoparticles offer another brain‐targeted carrier for cytoskeleton modulation. Lactoferrin‐PLGA and related ligands (transferrin, insulin, RVG) enhance BBB transcytosis of small‐molecule cytoskeletal drugs (ROCK/HDAC6 inhibitors) or RNA cargos and have reproducibly improved bioavailability in PD/AD models and intranasal routes (Zhang et al. 2021). Engineered nanoscale interfaces (nanotopographies, peptide amphiphiles) can bias actin waves and growth‐cone dynamics, suggesting future routes to locally guide mitochondrial positioning/fission near synapses or growth cones. These are early‐stage but provide a design space for instructive scaffolds that couple actin patterning to mitochondrial demand/supply (Pathak et al. 2025).

## 8 Discussion

Despite significant advances in understanding actin–mitochondria interactions in neurodegeneration, many questions remain unanswered. Mitochondrial dynamics and the actin cytoskeleton are tightly interwoven threads in the fabric of neuronal health. Over 2015–2025, substantial evidence has revealed that when this interplay goes awry, whether through cofilin–actin rod formation in Alzheimer's disease blocking mitochondrial transport (Wurz et al. 2022), α‐synuclein‐induced actin destabilization in Parkinson's disease fragmenting mitochondria (Ordonez et al. 2018), or loss of actin regulators like profilin‐1 in ALS leading to mitochondrial dysfunction (Read et al. 2024), the result is neuronal injury and neurodegeneration. Among these disorders, Alzheimer's disease has the most extensively documented actin–mitochondria pathology, making it a focal point for mechanistic studies. Parkinson's and other diseases reinforce and extend the concept, suggesting that actin‐mediated mitochondrial dysregulation is a common pathophysiological motif.

Furthermore, the role of actin in mitochondrial quality control pathways like mitophagy remains incompletely understood. Does actin remodeling facilitate the enclosure of damaged mitochondria by autophagosomes in neurons, and do disease proteins impede this? This is particularly relevant for PINK1/Parkin‐related PD and perhaps ALS (C9ORF72 has been linked to autophagy). Another open question is how early in the disease course do mitochondria‐actin disruptions occur? Are they initiators of degeneration or late‐stage amplifiers? Longitudinal studies in animal models and patients (e.g., via biomarkers of cytoskeletal injury or mitochondrial stress in CSF) could shed light on this. Additionally, we must consider other cytoskeletal elements: microtubules and intermediate filaments interact with actin and mitochondria (for instance, neurofilament aggregation in ALS can indirectly affect actin stability), suggesting that integrated cytoskeletal approaches may be needed.

The field still lacks detailed mechanistic understanding of mitochondria–ER contacts and actin nucleation at MAMs. The identities and regulation of the actin nucleators and adaptors that organize short F‐actin at OMM/MAMs remain poorly defined. Specifically, how disease proteins retune this nano‐architecture to bias Drp1 access and Ca^2+^/lipid exchange requires further investigation (Hatch et al. 2016; Lee et al. 2016). Additionally, the extent to which actin remodeling is required for PINK1/Parkin‐dependent versus receptor‐mediated mitophagy, and whether cofilin–actin rods impede autophagosome formation or cargo selection in neurites, is unresolved. A priority for the field is standardized methods for in vivo quantification of actin‐dependent mitochondria–ER contacts in human‐relevant neurons, coupled with causal perturbations and translational readouts. Recent evidence that organelle‐localized actin regulates both fission and fusion motivates spatiotemporal maps of actin nucleators and motors at MERCs in neurons. In parallel, maturing tools like SPLICS/MERBiT for live MERC measurements, optogenetic tethers for precise spatiotemporal control, and proximity/spatial proteomics for interactome mapping can reveal whether pathological drivers (Aβ/tau, α‐syn, mHTT, PFN1) derail specific checkpoints such as Drp1 recruitment, MFN2 capture, or matrix Ca^2+^ microdomains (Barazzuol et al. 2025; Gatti et al. 2025; Qiu et al. 2022).

On the therapeutic side, selective mitophagy enhancers and interface‐tuned cytoskeleton modulators should be paired with brain‐targeted, mitochondria‐addressed nanocarriers (Pathak et al. 2025; Pham et al. 2025), alongside rigorous localization and safety data (including CSF protein corona characterization and microglial responses). We also lack empiric boundaries for *how much*, *where*, and *for how long* to modulate actin in neurons to restore organelle function without impairing plasticity; most tools remain broad or off‐target (Nishimura et al. 2021). Finally, translating these insights to patients will require agents that selectively engage pathological cytoskeleton–mitochondria interactions without compromising their essential physiological roles. To address these gaps, live‐cell imaging of actin–mitochondria dynamics in neurons should be prioritized. This approach should track transient F‐actin assemblies, Drp1 recruitment/oligomerization, membrane potential, and Ca^2+^ at single‐organelle resolution in axons and spines. Complementary multi‐omics methods can map cytoskeletal changes to metabolic outcomes. For example, proximity‐labeling proteomics targeted to the OMM and MAMs, combined with metabolomics/flux assays and single‐cell or spatial transcriptomics, can link local cytoskeletal remodeling to bioenergetic rewiring. CRISPR‐based perturbation screens focused on actin regulators (cofilin, PFN1, formins) and Drp1 adaptors, read out by mitophagy and transport reporters, would help establish causal hierarchies. Achieving this balance between efficacy and safety is crucial for therapeutic development, given the systemic importance of actin and mitochondria in cellular function.

## Funding

This work was supported by the National Institutes of Health (CA267180, TL1 TR001858).

## Conflicts of Interest

The authors declare no conflicts of interest.

## Data Availability

The data that support the findings of this study are available from the corresponding author upon reasonable request.
